# Effect of HY7602-Fermented Deer Antler Extract on Selected Muscle-Related Outcomes and Fermentation-Associated Metabolomic Profiles

**DOI:** 10.4014/jmb.2604.04018

**Published:** 2026-07-15

**Authors:** Dong-Ki Hong, Soomin Jeon, Man Young Jo, Soo-Dong Park, Il-Dong Choi, Jae-Jung Shim, Do Yup Lee, Jae-Hwan Lee

**Affiliations:** 1 R&BD Center, hy Co., Ltd., 22, Giheungdanji-ro 24 beon-gil, Giheung-gu, Yongin-si 17086, Gyeonggi-do, Republic of Korea; 2 Department of Agricultural Biotechnology, Seoul National University, Seoul 08826, Republic of Korea

**Keywords:** *Latilactobacillus curvatus* HY7602, Deer antler extract, Fermentation, Metabolomics, Muscle-related outcomes

## Abstract

Age-related loss of muscle strength is closely associated with frailty, reduced mobility, and loss of independence. In this study, we investigated the clinical and metabolomic features of deer antler extract fermented with *Latilactobacillus curvatus* HY7602. A 12-week randomized, double-blind, placebo-controlled trial was conducted in adults with reduced muscle function to evaluate the effects of daily intake of the fermented extract on muscle-related outcomes and functional performance. In parallel, exploratory untargeted LC-MS/MS-based metabolomic profiling was performed to examine biochemical changes during fermentation at three stages: extract (E), HY7602-supplemented extract (L), and fermented extract (F). After 12 weeks, the fermented deer antler extract group showed improvements in selected muscle-related outcomes compared with the placebo group, including bilateral and unilateral hand grip strength, right quadriceps strength, and Short Physical Performance Battery chair-stand performance. Metabolomic profiling revealed stage-dependent differences among the three preparation stages. Principal component analysis showed distinct clustering of E, L, and F, with the largest separation observed between E and F. Trajectory analysis suggested progressive changes in polyamine-related metabolites, amino acid derivatives, and γ-glutamyl peptides. Pathway analysis further indicated coordinated differences in nitrogen and amine metabolism, polyamine metabolism, glutathione-related metabolism, nucleotide metabolism, and central carbon metabolism. HY7602-fermented deer antler extract may support selected strength-related outcomes in adults with reduced muscle function. Metabolomic profiling characterized the fermented product and provides a foundation for future mechanistic studies.

## Introduction

Age-related decline in skeletal muscle strength is a major contributor to frailty, reduced mobility, and loss of independence in older adults [1, 2]. Reduced muscle function is closely associated with poorer physical performance and a higher risk of disability in later life, making preservation of muscle strength an important target for healthy aging [3-5]. Although exercise remains the primary strategy for preventing or attenuating sarcopenia, nutritional interventions and probiotic-related approaches have increasingly been explored as complementary strategies to support muscle function during aging [6-9].

Deer antler has long been used as a traditional bioactive material and contains diverse constituents, including peptides, amino acids, lipids, and other low-molecular-weight compounds that may contribute to its biological activity [10, 11]. Recent experimental studies have suggested that deer antler extract may influence muscle-related outcomes, including myogenic differentiation and protection against muscle atrophy in cell models [12]. However, as with many complex natural materials, its functional properties are likely determined not by a single compound but by the overall biochemical composition of the extract.

Fermentation is increasingly recognized as an effective strategy for improving the functional properties of food-derived materials by reshaping their chemical composition through microbial biotransformation [13-15]. During fermentation, microorganisms can release, convert, or newly generate metabolites that contribute to changes in the nutritional, functional, and sensory characteristics of the final product [16, 17]. In particular, lactic acid bacteria with favorable fermentation properties, including *Latilactobacillus curvatus*, have attracted attention for their potential to enhance the value of bioactive materials through controlled fermentation [18].

Previous in vitro and *in vivo* studies have suggested that HY7602-fermented deer antler may improve muscle-related phenotypes, including endurance capacity, mitochondrial biogenesis, grip strength, muscle mass, muscle strength, resistance to dexamethasone-induced atrophy, and fatigue-related outcomes [19-22]. These findings provide a biological rationale for evaluating HY7602-fermented deer antler in humans. However, previous studies primarily focused on biological responses and provided limited information regarding the fermentation-associated compositional changes occurring in the material itself.

This represents an important gap because the compositional consequences of fermentation cannot be fully understood without defining how HY7602-mediated fermentation remodels the biochemical composition of deer antler extract. In other words, identifying the metabolites and metabolic domains altered by fermentation is necessary to explain why fermentation was applied, to understand the compositional differences between fermented and non-fermented preparations, and to interpret preclinical and clinical findings within a common product-level biochemical framework. Untargeted metabolomics is particularly useful for this purpose because it enables comprehensive characterization of fermentation-associated compositional remodeling across multiple classes of metabolites [23-27].

Therefore, the present study combined a 12-week randomized, double-blind, placebo-controlled clinical trial with untargeted LC-MS/MS-based metabolomic profiling. The clinical trial evaluated selected muscle-related outcomes in adults with reduced muscle function, whereas the metabolomic analysis characterized stage-dependent compositional changes across extract (E), HY7602-supplemented extract (L), and fermented extract (F). This design was intended to define the biochemical consequences of HY7602-mediated fermentation and to provide a product-level framework for interpreting fermentation-associated compositional changes.

## Materials and Methods

### Ethics and Study Design

This study was designed as a 12-week, randomized, double-blind, placebo-controlled, multicenter clinical trial with two parallel groups, in which participants received either fermented antler (FA) or placebo in pouch formulations. The trial was conducted at Chung-Ang University Gwangmyeong Hospital and Chungnam National University Sejong Hospital in South Korea between 2022 and 2023.

The study protocol was approved by the Institutional Review Boards of Chung-Ang University Gwangmyeong Hospital (IRB No. 2205-004-004) and Chungnam National University Sejong Hospital (CNUSH 2022-06-008). All procedures were conducted in accordance with the Declaration of Helsinki and the Korean Good Clinical Practice (KGCP) guidelines. The trial was registered at the Korean Clinical Research Information Service (KCT0011733, https://cris.nih.go.kr/). The sample size was calculated based on the expected difference in quadriceps muscle strength between groups, assuming 80% statistical power and a significance level of 0.05. All participants provided written informed consent prior to enrollment. Screening assessments were conducted to determine eligibility, and participants who met the inclusion criteria were enrolled within two weeks of the screening visit. Eligible participants were randomly assigned to either the FA or placebo group and ingested the assigned product once daily for 12 weeks. Participants visited the study sites every six weeks during the intervention period for evaluation of efficacy outcomes, vital signs, medication history, changes in medical condition, and monitoring of adverse events.

### Subjects

All participants were informed about the purpose, procedures, and potential risks of the study prior to enrollment.

**Inclusion criteria:**

1. Adults aged 50–85 years.

2. Individuals with reduced hand grip strength (<36 kg for men and <22 kg for women).

3. Participants with skeletal muscle mass below 100% of the standard reference range measured using InBody bioelectrical impedance analysis.

4. Individuals who voluntarily agreed to participate and provided written informed consent.

**Exclusion criteria:**

1. Individuals unable to perform physical activity due to musculoskeletal disorders, neurological disorders, or cognitive impairment, including frailty-related hip fractures.

2. Body mass index (BMI) ≥ 30 kg/m^2^.

3.Individuals who had received systemic steroid therapy or hormone therapy within four weeks prior to screening, or who had taken health supplements or muscle-strengthening supplements within two weeks prior to screening.

4. Any other condition deemed inappropriate for participation by the investigators.

### Preparation of Fermented Deer Antler Extract

Fermented deer antler extract was prepared as previously described [19]. Briefly, hot air-dried deer antler (Cervus elaphus Linné) was sliced and extracted with distilled water (1:30, w/w) at 95 ± 2°C, followed by filtration. The extract was supplemented with freeze-dried HY7602 culture (1%, w/w; ≥1.0 × 10^10^ CFU/g) and fermented at 37 ± 2°C for 24 ± 1 h. After fermentation, the product was pasteurized, concentrated, lyophilized with maltodextrin, and stored at 1–35°C until use.

### Intervention

Participants who met the eligibility criteria were randomly assigned to either the FA group or placebo group in a 1:1 ratio.

At baseline, all participants completed baseline assessments, including demographic and lifestyle surveys, medical and medication history review, physical examination, vital signs, anthropometric measurements, clinical laboratory tests, pregnancy testing (for women of childbearing potential), electrocardiography, skeletal muscle mass assessment using InBody, hand grip strength measurement, and blood biomarker analysis.

All investigational products were supplied by hy Co., Ltd. (Republic of Korea). Participants consumed one pouch (80 mL/day) of the assigned product orally each morning for 12 weeks. The FA product contained 580 mg/day of fermented antler extract powder in an 80 mL pouch formulation. The placebo product was manufactured to be identical in appearance, taste, and packaging to maintain the double-blind design.

Participants were instructed to maintain their habitual diet and physical activity levels throughout the intervention period. To minimize confounding factors, participants were advised to avoid excessive alcohol consumption and vigorous exercise during the study period, particularly during the week preceding each study visit. Participants were encouraged to perform moderate-intensity walking (30–60 min per session, at least three times per week).

### Outcome Measures

**Quadriceps strength.** Quadriceps muscle strength was assessed using an isokinetic dynamometer (Con-Trex). Measurements were performed at an angular velocity of 60°/s. Each test consisted of five repetitions with a 30-sec rest interval between trials, and the average of three valid trials was used for analysis.

**Grip strength.** Hand grip strength was measured using a hand dynamometer while participants were seated. Each participant performed three maximal attempts, and the highest value was recorded.

**Short Physical Performance Battery (SPPB).** The Short Physical Performance Battery (SPPB) was used to assess lower extremity physical function [28]. The SPPB consists of three components: standing balance, 4-meter gait test, and repeated chair stands. In the present study, each SPPB component was analyzed separately.

**Blood markers and oxidative stress measurements.** Blood samples were collected after overnight fasting and analyzed at a certified clinical laboratory. Serum levels of creatine kinase (CK), lactate dehydrogenase (LDH), AST, ALT, blood urea nitrogen (BUN), and creatinine were measured using an automated biochemical analyzer. Inflammatory cytokines (TNF-α, IL-1β, and IL-6) were quantified using enzyme-linked immunosorbent assay (ELISA) kits according to the manufacturer’s instructions. Antioxidant markers including glutathione (GSH) and superoxide dismutase (SOD) were determined using commercially available assay kits.

**Compliance and safety evaluation.** Compliance was calculated as: Compliance (%) = (Number of products consumed ÷ Number prescribed) × 100. Safety assessments included physical examination, clinical laboratory tests, ECG, and monitoring of adverse events.

### Statistical Analysis for Clinical Data

Statistical analyses were performed using SAS software (version 9.4). Statistical significance was defined as *p* < 0.05 (two-tailed). Within-group comparisons were analyzed using paired t-tests, and between-group comparisons were performed using two-sample t-tests or Wilcoxon rank-sum tests depending on normality assessed using the Shapiro–Wilk test. When baseline differences existed, a Generalized Linear Model (GLM) was applied with baseline variables included as covariates.

### LC-MS/MS Metabolomics Analysis

Untargeted LC-MS/MS profiling was performed on three independent biological replicates for each stage, including extract (E), HY7602-supplemented extract (L), and fermented extract (F) (n = 3 per stage). Given the limited number of biological replicates, the metabolomic analysis was intended as an exploratory characterization of fermentation-associated compositional changes.

**Sample preparation for LC-MS/MS.** Untargeted metabolomic profiling was conducted to characterize metabolic alterations during the fermentation process. Each sample (100 μL) was transferred into 2.0 mL safe-lock microtubes (Eppendorf, Germany). Metabolites were extracted using 1600 μL of extraction solvent consisting of methanol, isopropanol, and water (3:3:2, v/v/v). Samples were homogenized using a Mixer Mill (28 Hz for 60 s; Retsch GmbH & Co., Germany), followed by sonication on ice for 10 min. The homogenized samples were centrifuged at 13,200 rpm at 4°C for 10 min. Approximately 750 μL of the supernatant was transferred to new 1.5 mL microtubes and completely dried using a vacuum concentrator (ScanVac, Republic of Korea). The dried extracts were reconstituted in 100 μL of distilled water prior to LC-MS/MS analysis.

**LC–Orbitrap MS analysis.** Metabolite profiling was performed using a Vanquish ultra-performance liquid chromatography (UPLC) system coupled with a Q-Exactive Focus Orbitrap mass spectrometer (Thermo Fisher Scientific, USA). Chromatographic separation was achieved using an Acquity UPLC BEH C18 column (2.1 × 100 mm, 1.7 μm; Waters, USA) equipped with a BEH C18 VanGuard pre-column (2.1 × 5 mm, 1.7 μm). The mobile phase consisted of solvent A (0.1% formic acid in water) and solvent B (0.1% formic acid in acetonitrile). Metabolites were eluted using a binary gradient system. The mass spectrometer was operated in both positive and negative electrospray ionization (ESI) modes to expand metabolite coverage.

Quality control (QC) samples were prepared by pooling aliquots from all samples and injected periodically throughout the analytical sequence to monitor instrument stability and analytical reproducibility.

**Metabolomics data processing.** Raw LC-MS/MS data files were processed using MS-DIAL software (version 4.9) [29]. Peak detection, deconvolution, alignment, and metabolite annotation were performed within the MS-DIAL workflow.

The data processing parameters were as follows:

• MS1 mass range: 80–1200 Da

• Minimum peak height: 50,000 amplitude

• Retention time tolerance (alignment): 0.2 min

• MS1 tolerance: 0.005 Da

• MS2 tolerance: 0.01 Da

Metabolite identification was performed using both an in-house metabolite library and publicly available MS/MS spectral libraries included in MS-DIAL. Retention time filtering of 0.5 min was applied for identification using the in-house library. A similarity score above 70% was used as the threshold for metabolite annotation. Features detected in less than 50% of samples in at least one experimental group were excluded from further analysis. Blank filtering was also applied using a five-fold threshold based on the ratio between the maximum sample signal and the average blank signal. Prior to statistical analysis, metabolite peak intensities were normalized to the total signal intensity of each sample. A scaling factor based on the ratio of the mean total intensity across all samples to the total intensity of each individual sample was applied to correct for systematic variation among samples.

**Multivariate statistical analysis.** The processed metabolomics dataset was normalized prior to statistical analysis. Principal component analysis (PCA) was first performed as an unsupervised method to visualize global compositional variation among extract (E), HY7602-supplemented extract (L), and fermented extract (F) samples [30]. Permutational multivariate analysis of variance (PERMANOVA) was additionally performed using the normalized metabolomic dataset to assess group-wise differences among E, L, and F samples, with 999 permutations. The results were reported as the F statistic, R^2^ value, and permutation-based *p*-value.

**Volcano plot analysis.** Volcano plot analyses were performed as an exploratory feature-screening approach for pairwise comparisons among the three preparation stages: F vs. E, L vs. E, and F vs. L. For each comparison, log2 fold change was calculated for each metabolite feature, and two-tailed t-tests were performed. False discovery rate-adjusted *q*-values were calculated using the Benjamini–Hochberg procedure. Features with an absolute log2FC > 1 and an FDR-adjusted *q*-value < 0.05 were considered differential metabolite features.

**Pattern Hunter analysis.** Pattern-based metabolite analysis was conducted using the Pattern Hunter algorithm implemented in MetaboAnalyst 6.0 [30]. This analysis was used to identify metabolites exhibiting specific expression patterns across fermentation stages (E → L → F). Metabolites showing monotonic increasing or decreasing patterns were identified based on correlation with predefined pattern vectors.

**Heatmap visualization.** Hierarchical clustering analysis and heatmap visualization were performed to illustrate relative metabolite abundance patterns across samples. Metabolite intensities were normalized and scaled using Z-score transformation. Clustering was conducted using Euclidean distance and Ward’s linkage method. The resulting heatmaps were used to visualize metabolite clustering and group-specific metabolic signatures.

**Representative metabolite analysis.** Representative metabolites were selected from significantly altered metabolites associated with enriched metabolic pathways to illustrate stage-dependent metabolic changes during HY7602-mediated fermentation. Differences among extract (E), HY7602-supplemented extract (L), and fermented extract (F) were assessed using the Kruskal–Wallis test, followed by pairwise multiple comparisons. *P*-values from pairwise comparisons were adjusted using the Benjamini–Hochberg false discovery rate (FDR) method. Because this representative metabolite analysis was exploratory and based on a limited number of biological replicates, comparisons with *q* < 0.10 were considered to meet an exploratory FDR threshold for visualization and interpretation.

**Pathway enrichment and network visualization.** Pathway enrichment analysis was performed using MetaboAnalyst 6.0 based on KEGG pathway annotations [30, 31]. Differential or stage-dependent metabolites identified from univariate and pattern-based analyses were used as input for pathway analysis. Over-representation analysis and pathway topology analysis were applied to identify significantly enriched metabolic pathways. Statistical significance was evaluated using false discovery rate (FDR)-adjusted *p*-values, and pathway impact values were calculated based on topology-derived centrality measures. The resulting enriched pathways and associated metabolites were visualized as a metabolite–pathway interaction network using Cytoscape (version 3.10.4) [32].

## Results

### Participant Flow

**Fig. 1:**
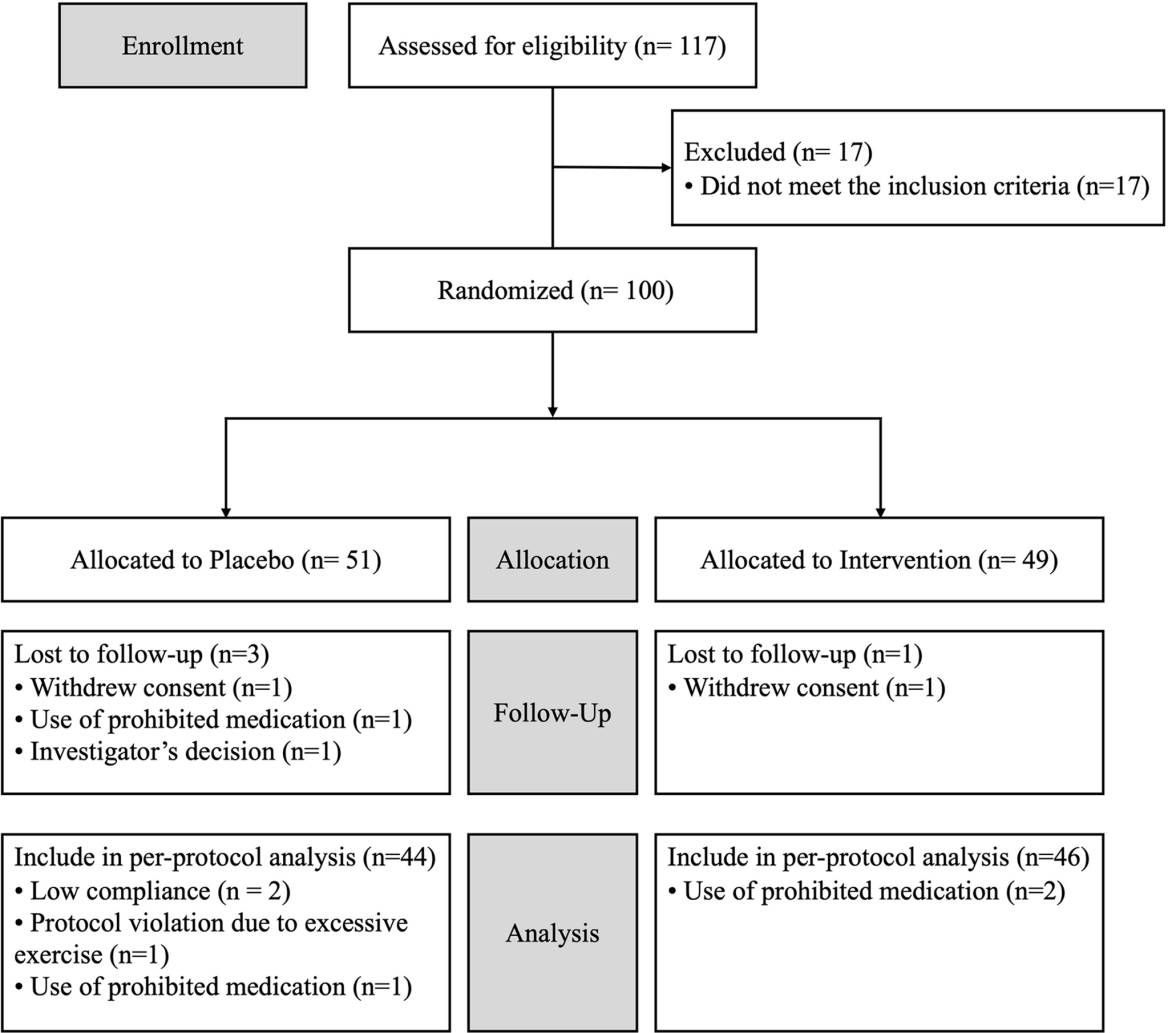
CONSORT flow diagram of participant recruitment, allocation, follow-up, and analysis in the randomized, double-blind, placebocontrolled clinical trial.

A total of 117 individuals were assessed for eligibility, of whom 17 were excluded for not meeting the inclusion criteria. The remaining 100 participants were randomized to the placebo group (n = 51) or the FA group (n = 49). During the intervention period, three participants in the placebo group and one participant in the intervention group were lost to follow-up. After excluding participants with low compliance or protocol violations, 44 participants in the placebo group and 46 participants in the intervention group were included in the final per-protocol analysis (Fig. 1).

### Clinical Outcomes

**Fig. 2:**
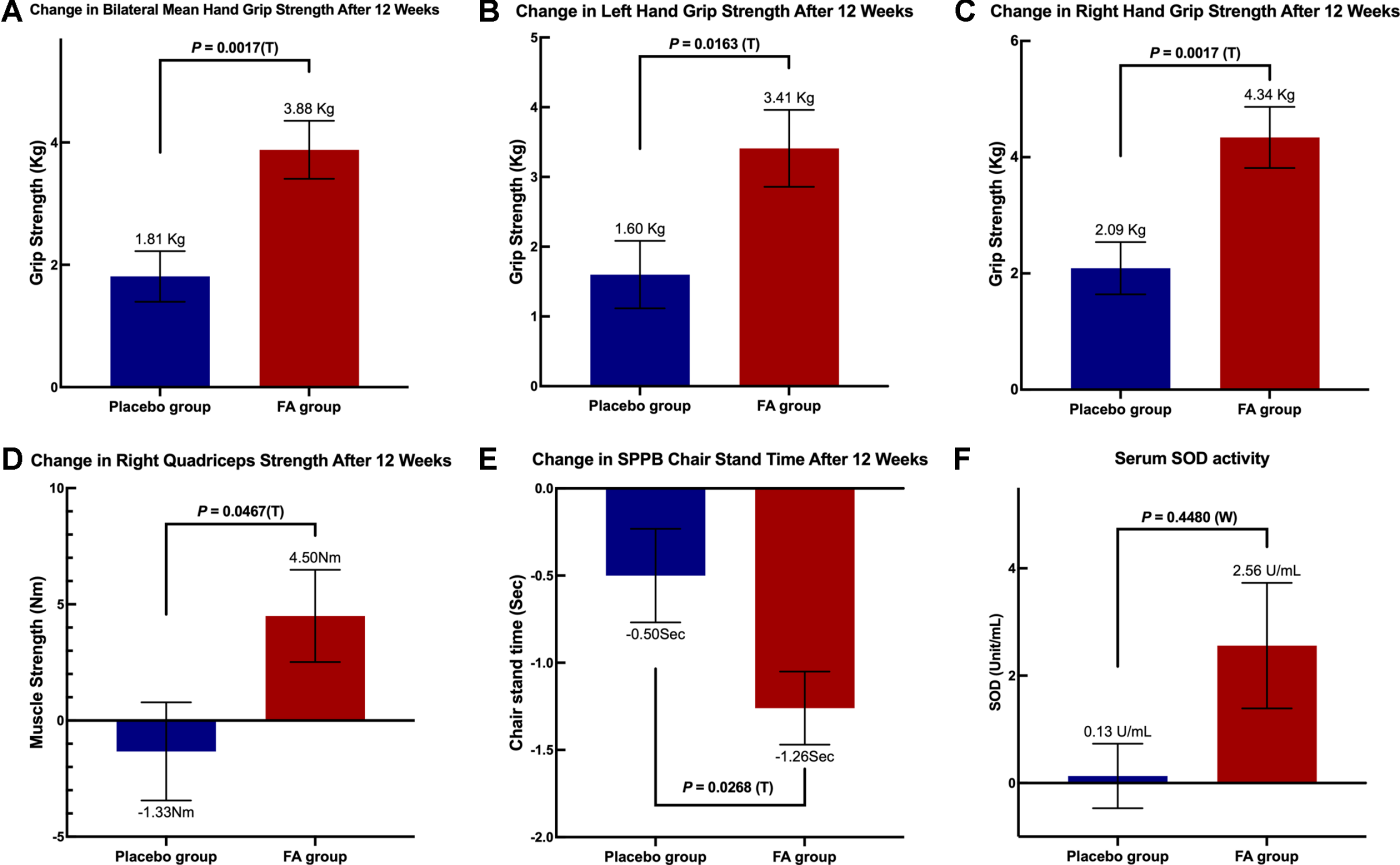
Effects of 12-week intake on muscle strength, functional performance, and antioxidant activity. Bar graphs represent mean ± SEM changes from baseline in the placebo and treatment groups. Significant improvements were observed in bilateral and unilateral hand grip strength (**A–C**), right quadriceps strength (**D**), and SPPB chair-stand performance (**E**) in the treatment group compared with placebo. Serum SOD activity (**F**) showed a numerical increase but did not reach statistical significance. *P*-values were calculated using independent two-tailed t-tests (**T**) or Wilcoxon rank-sum tests (**W**), as indicated in each panel.

Baseline demographic and general characteristics of the participants are summarized in Supplementary Table 1, with no meaningful differences observed between the FA and placebo groups. As shown in Fig. 2, after 12 weeks, the FA group showed greater increases than the placebo group in bilateral mean hand grip strength (3.88 ± 0.47 vs. 1.81 ± 0.41 kg, *p* = 0.0017), left hand grip strength (3.41 ± 0.55 vs. 1.60 ± 0.48 kg, *p* = 0.0163), and right hand grip strength (4.34 ± 0.52 vs. 2.09 ± 0.45 kg, *p* = 0.0017). Among lower-extremity outcomes, between-group differences were observed for right quadriceps strength (4.50 ± 1.98 vs. -1.33 ± 2.11 N·m, *p* = 0.0467) and chair-stand time (-1.26 ± 0.21 vs. -0.50 ± 0.27 s, *p* = 0.0268). In contrast, left quadriceps strength (3.92 ± 2.02 vs. 2.39 ± 2.43 N·m, *p* = 0.6286), SPPB total score (0.20 ± 0.07 vs. 0.00 ± 0.09, *p* = 0.0714), 4-m gait time (-0.20 ± 0.06 vs. -0.17 ± 0.06 s, *p* = 0.6834), and SOD (2.56 ± 1.17 vs. 0.13 ± 0.60 U/mL, *p* = 0.4480) did not differ significantly between groups. Detailed outcome data, including baseline and week 12 values, change from baseline, and within-group and between-group comparisons, are provided in Supplementary Table 2. Safety outcomes are summarized in Supplementary Table 3. The incidence and overall pattern of adverse events (AEs) were comparable between the FA and placebo groups. Most AEs were mild in severity, and no severe events or discontinuations related to AEs were reported. Causality assessments did not indicate any clear relationship between the intervention and the observed AEs.

### Multivariate Analysis of Metabolomic Profiles

**Fig. 3:**
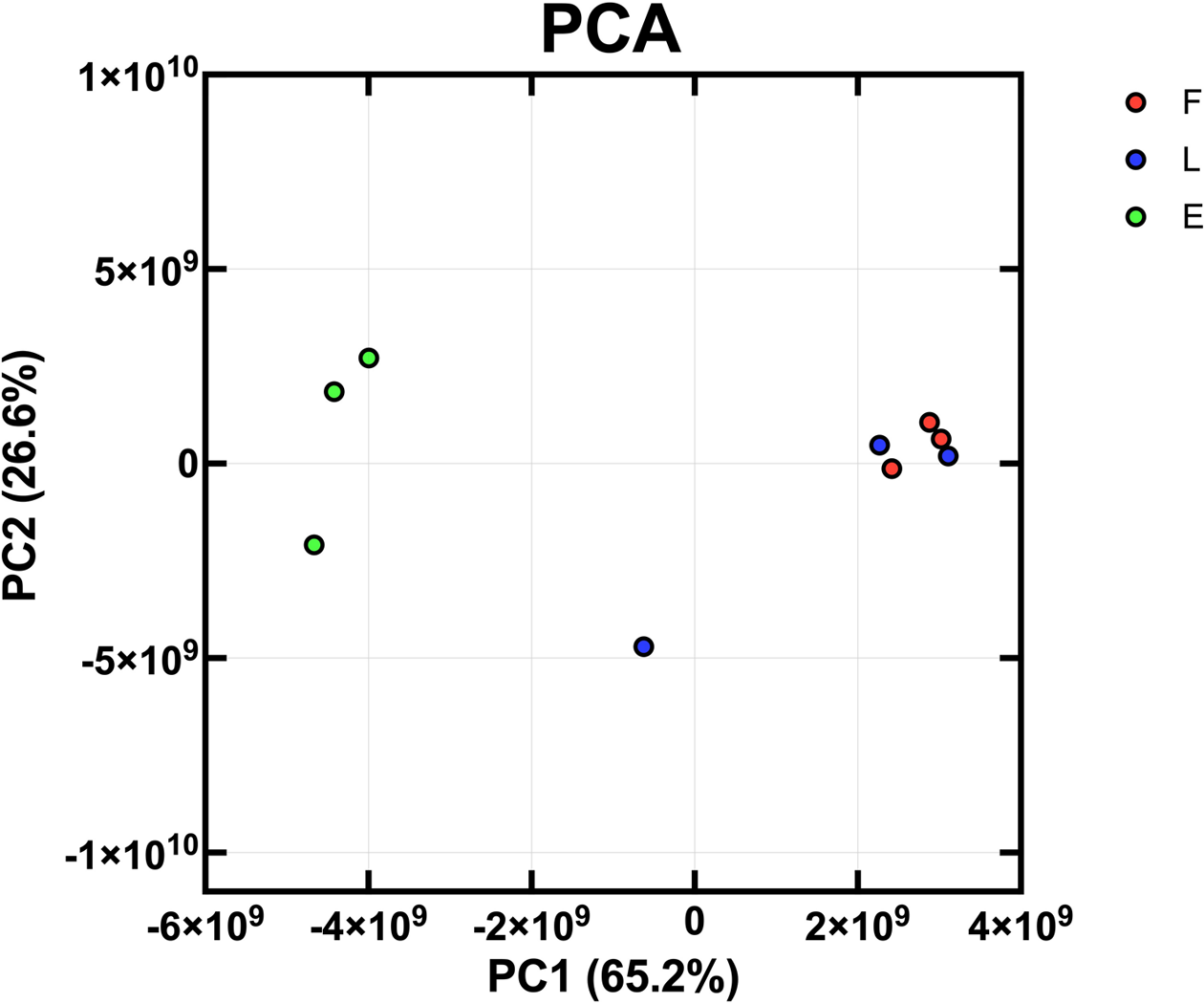
Principal component analysis (PCA) score plot of metabolomic profiles across preparation stages. PCA score plot of normalized LC-MS/MS metabolomic data showing the distribution of extract (E), HY7602-supplemented extract (L), and fermented extract (F) in reduced principal component space. Each point represents a biological replicate (n = 3). The first two principal components explained 65.2% (PC1) and 26.6% (PC2) of the total variance. Samples are separated primarily along PC1, which distinguishes the extract group from the fermentation-associated groups (L and F), while PC2 contributes to additional variation among samples. Group-wise differences in overall metabolomic composition were supported by PERMANOVA (F = 7.392, R^2^ = 0.711, *p* = 0.015; 999 permutations).

Principal component analysis (PCA) was performed to evaluate global metabolic differences among extract (E), HY7602-supplemented extract (L), and fermented extract (F). As shown in Fig. 3, the first two principal components explained 91.8% of the total variance (PC1: 65.2%; PC2: 26.6%). PC1 clearly separated the extract group from the fermentation-associated groups (L and F), indicating that bacterial supplementation and fermentation induced substantial metabolic variation. Extract samples clustered distinctly on the negative side of PC1, whereas L and F samples were positioned on the positive axis. Fermented samples were clustered separately from L, suggesting further metabolic divergence during active fermentation. Biological replicates within each group demonstrated tight clustering, supporting analytical reproducibility.

### Volcano Plot Analysis of Differential Features

**Fig. 4:**
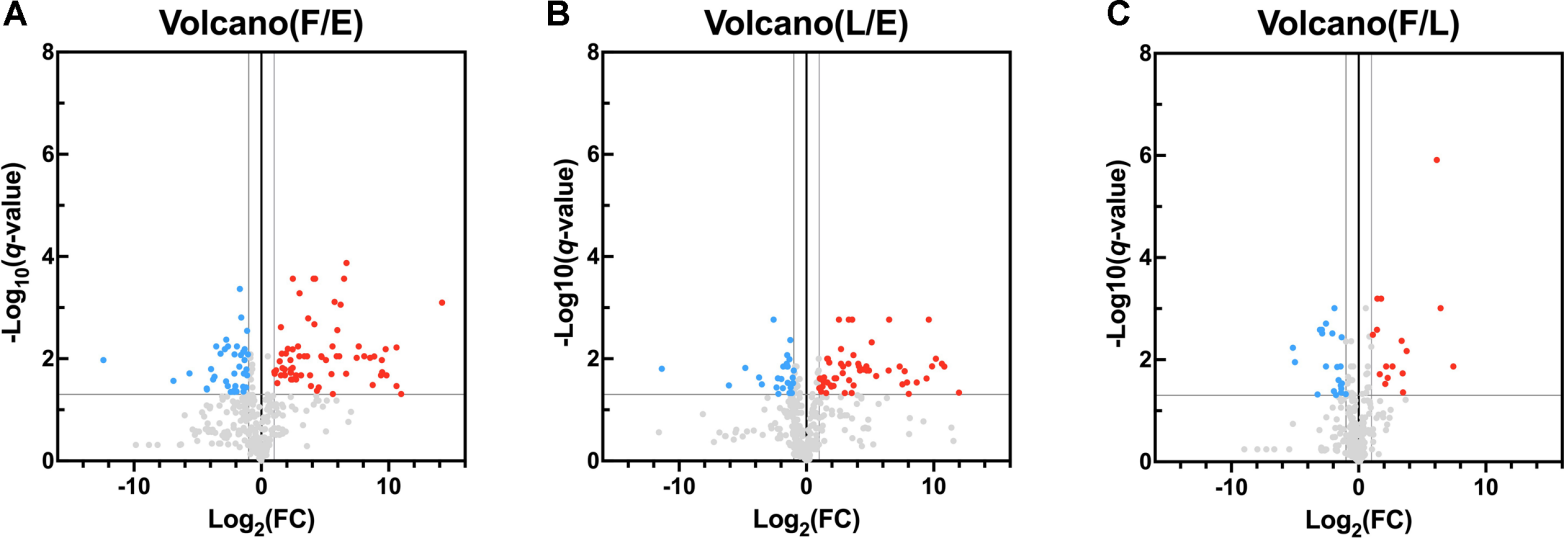
Volcano plot analysis of differential metabolites among experimental groups. Volcano plots illustrate the relationship between log2 fold change (log_2_FC) and −log_10_(*q*-value) for pairwise comparisons among extract (E), HY7602-supplemented extract (L), and fermented extract (F): (**A**) F vs. E, (**B**) L vs. E, and (**C**) F vs. L. Differential metabolites were identified using two-tailed t-tests, with thresholds of |log_2_FC| > 1 and false discovery rate (FDR)-adjusted *q*-values < 0.05 based on the Benjamini–Hochberg procedure. Each point represents an individual metabolite feature. Metabolites that met both significance criteria are highlighted in color, with red indicating upregulated features and blue indicating downregulated features in each comparison, while gray points represent non-significant features. Vertical lines denote the fold-change threshold (|log_2_FC| = 1), and the horizontal line indicates the significance threshold used for visualization.

Untargeted LC-MS/MS profiling followed by exploratory univariate screening suggested preparation-stage differences in metabolite abundance among extract (E), HY7602-supplemented extract (L), and fermented extract (F) (Fig. 4). Using the prespecified screening criteria (absolute log2FC > 1 and FDR-adjusted *q* < 0.05), the E-to-F comparison indicated a relative increase in feature abundance in the fermented samples. In contrast, the E-to-L comparison showed a transitional pattern, while the L-to-F comparison reflected continued redistribution of features, suggesting progressive compositional changes during fermentation. Overall, these patterns indicate compositional divergence between the initial extract and the fully fermented material, with intermediate changes observed following HY7602 supplementation.

### Heatmap Analysis of Metabolites Differing Across Fermentation Stages

**Fig. 5:**
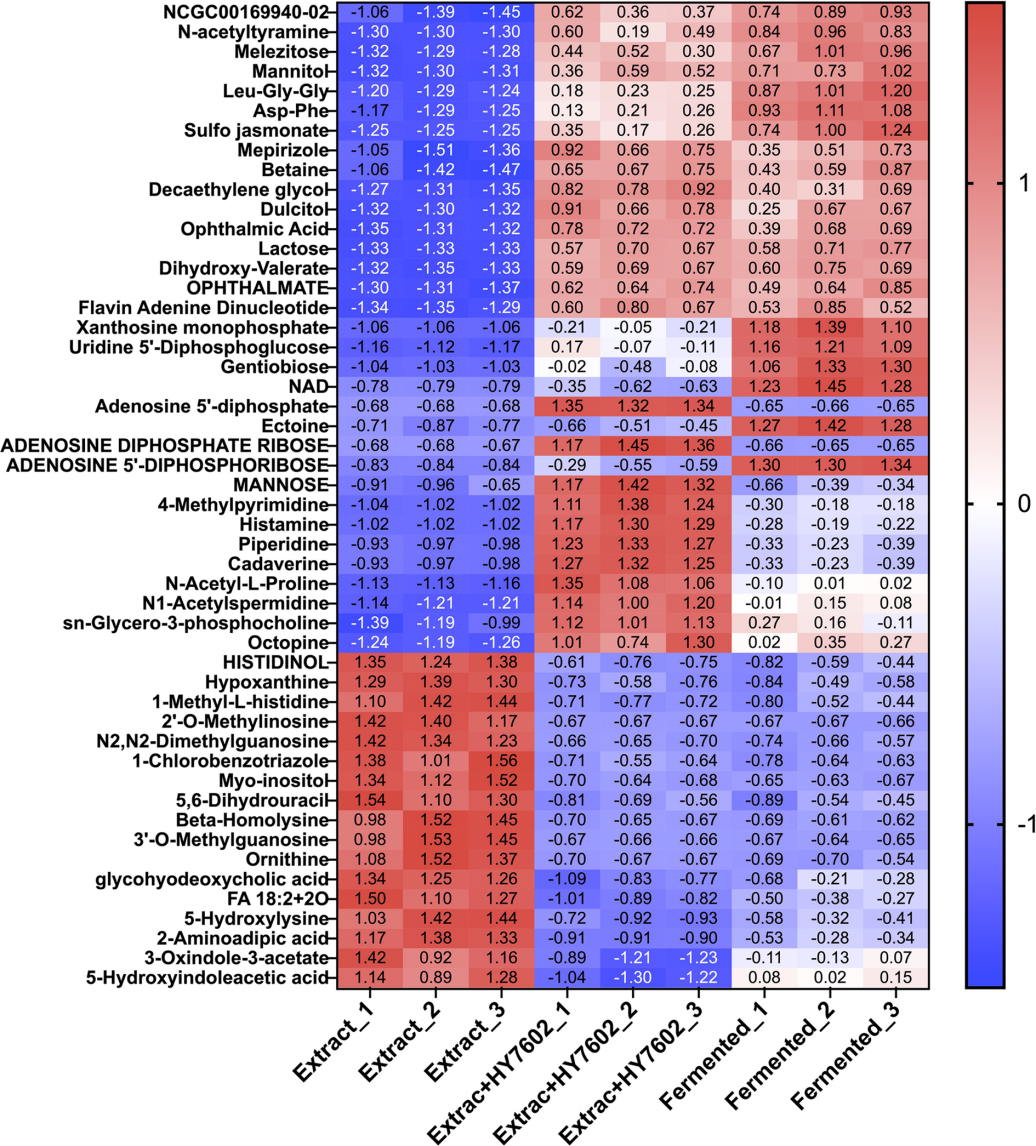
Heatmap analysis of stage-dependent metabolites. Heatmap visualization of selected metabolites across experimental groups. Row-wise z-score normalization (mean-centered and scaled by standard deviation) was applied to highlight relative abundance differences among samples. Blue and red colors indicate relatively decreased and increased metabolite levels, respectively. Extract (E), HY7602-supplemented extract (L), and fermented extract (F) groups include three biological replicates per group.

To visualize relative metabolite abundance patterns across experimental groups, a heatmap was generated using row-wise z-score normalization (mean-centered and scaled by standard deviation). As shown in Fig. 5, distinct clustering patterns were observed among extract (E), HY7602-supplemented extract (L), and fermented extract (F) samples.

Metabolites identified by Pattern Hunter as exhibiting a monotonic trajectory (E→L→F) demonstrated consistent relative enrichment in the fermented group. These features showed negative z-scores (blue) in E, intermediate levels in L, and positive z-scores (red) in F, indicating incremental enrichment from E to F.

Conversely, a subset of metabolites displayed inverse trajectories, characterized by relatively high abundance in E and reduced levels in F. The bidirectional modulation suggests that fermentation induces selective metabolic remodeling rather than uniform amplification.

Biological replicates within each group exhibited high intra-group consistency, supporting reproducibility of the fermentation-induced metabolic shift.

### Pattern Hunter Analysis of Stage-Dependent Trajectories

To investigate dynamic metabolic changes across fermentation stages, pattern-based analysis was performed using the Pattern Hunter module in MetaboAnalyst. A predefined monotonic increasing template (E < L < F) was applied to identify metabolites exhibiting progressive elevation from extract (E) to HY7602-supplemented extract (L), and further to fermented extract (F).

**Fig. 6:**
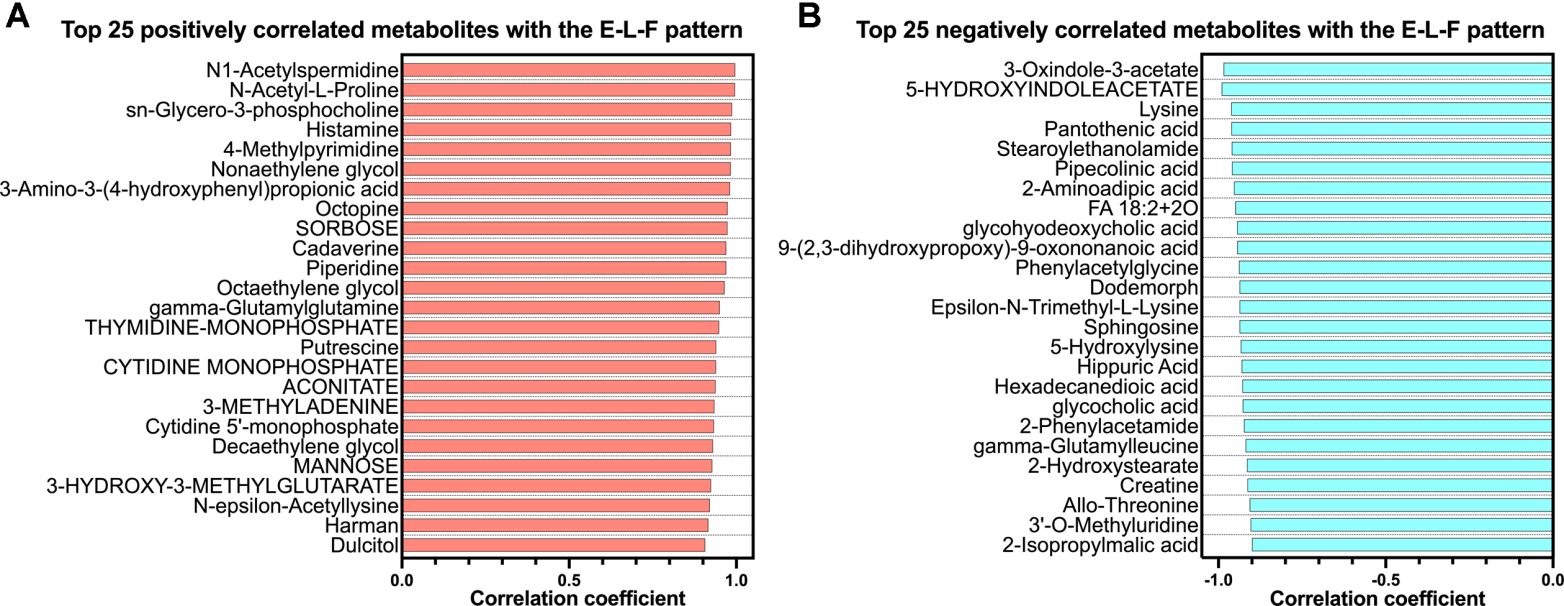
Pattern-based identification of metabolites exhibiting directional trajectories during fermentation. (**A**) Metabolites showing a monotonic increasing pattern (E < L < F). (**B**) Metabolites showing a monotonic decreasing pattern (E > L > F). Metabolites were analyzed using the Pattern Hunter module in MetaboAnalyst with predefined increasing and decreasing templates. Features were ranked based on Pearson correlation coefficients with the template pattern, and the top-ranked metabolites were selected for visualization. Data were obtained from three biological replicates per group (n = 3). Panel (A) summarizes metabolites progressively enriched from extract (E) to HY7602-supplemented extract (L) and to fermented extract (F), whereas panel (B) presents metabolites showing sequential depletion across the same stages. E, extract; L, HY7602-supplemented extract; F, fermented extract.

Pattern Hunter analysis identified a subset of metabolites strongly correlated with the predefined increasing trajectory (Fig. 6). These metabolites displayed consistent directional changes across fermentation stages, indicating structured metabolic progression rather than isolated pairwise differences.

The observed pattern suggests that metabolic transformation begins upon bacterial supplementation and continues during active fermentation, supporting the hypothesis of cumulative microbial biotransformation.

### Representative Metabolites Altered from Extract to Fermented Preparation

**Fig. 7:**
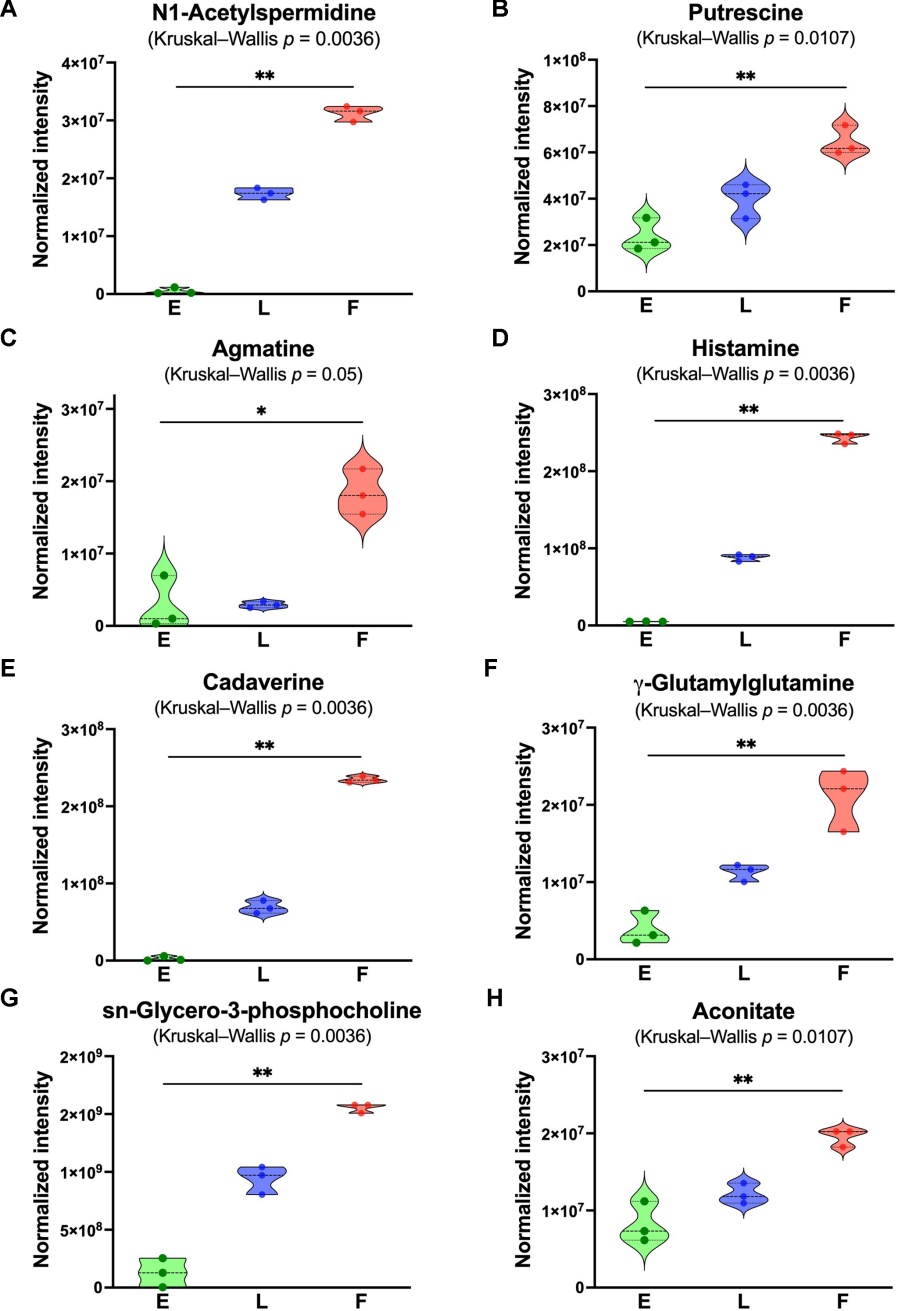
Representative metabolites exhibiting stage-dependent changes during HY7602-mediated fermentation. Violin plots present normalized LC-MS/MS intensities of selected metabolites in extract (E), HY7602-supplemented extract (L), and fermented extract (F). The metabolites shown are related to polyamine metabolism (**A**, N1-acetylspermidine; **B**, putrescine), nitrogen and amine metabolism (**C**, agmatine; **D**, histamine; **E**, cadaverine), glutathioneassociated redox metabolism (**F**, γ-glutamylglutamine), membrane-associated phospholipid remodeling (**G**, sn-glycero-3-phosphocholine), and central carbon metabolism (**H**, aconitate). Each group consisted of three biological replicates (n = 3). Statistical differences among groups were assessed using the Kruskal–Wallis test, followed by pairwise multiple comparisons. *P*-values from pairwise comparisons were adjusted using the Benjamini–Hochberg false discovery rate (FDR) method. A *q* < 0.10 threshold was used as an exploratory FDR criterion for representative metabolite visualization. Asterisks indicate pairwise differences meeting the exploratory FDR threshold (*q < 0.10, **q < 0.05).

Representative metabolite analysis further supported stage-dependent metabolic remodeling during HY7602-mediated fermentation (Fig. 7). These representative metabolites were associated with fermentation-related biochemical processes, including polyamine metabolism, nitrogen and amine metabolism, glutathione-related redox metabolism, membrane phospholipid remodeling, and central carbon metabolism. Overall, these metabolite patterns indicate product-level compositional changes associated with multiple fermentation-related pathways. Detailed annotation confidence information for representative and pathway-related metabolites is provided in Supplementary Table 4.

### Pathway Network Analysis

**Fig. 8:**
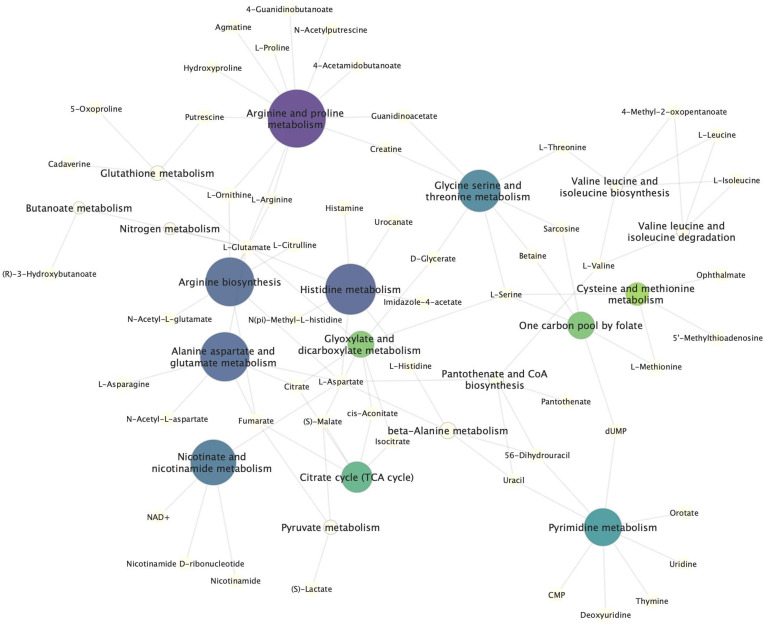
KEGG-based pathway enrichment network visualized using Cytoscape. Pathway enrichment analysis was performed using KEGG annotations based on metabolites identified from differential and stage-dependent analyses. False discovery rate (FDR)–adjusted *p*-values and pathway impact scores (topologybased centrality analysis) were calculated to identify significantly enriched pathways. Enriched pathways were visualized as a metabolite–pathway interaction network using Cytoscape (v3.10.4). Nodes represent metabolic pathways and associated metabolites, while edges indicate biochemical relationships derived from KEGG mapping. Node size corresponds to pathway impact value, and node color intensity reflects statistical significance based on FDR values. The network highlights coordinated remodeling of arginine and proline metabolism, glutathione metabolism, pyrimidine metabolism, nitrogen metabolism, and the citrate cycle (TCA cycle) during HY7602-mediated fermentation. E, extract; L, HY7602-supplemented extract; F, fermented extract.

To investigate pathway-level relationships among stage-dependent metabolites, pathway network analysis was performed (Fig. 8). The network revealed that fermentation-associated metabolites were enriched in interconnected pathways primarily related to amino acid metabolism, nitrogen metabolism, polyamine biosynthesis, nucleotide metabolism, and energy pathways.

Central nodes included arginine and proline metabolism, glutathione metabolism, pyrimidine metabolism, and the citrate cycle (TCA cycle). Metabolites such as L-ornithine, L-citrulline, putrescine, agmatine, N-acetylputrescine, cadaverine, and creatine were prominently associated with nitrogen flux and polyamine-related pathways.

Redox-related metabolites including NAD+, 5-oxoproline, and glutathione-linked intermediates were integrated within the same network, suggesting coordinated changes in redox-related metabolism during fermentation.

The network topology suggests that fermentation was accompanied by structured pathway-level remodeling rather than isolated metabolite changes.

## Discussion

This study evaluated HY7602-fermented deer antler extract by combining a 12-week randomized, double-blind, placebo-controlled clinical trial with untargeted LC-MS/MS-based product-level metabolomic profiling. These two components were conducted as complementary approaches to evaluate the same HY7602-fermented material from both a host-response perspective and a product-level biochemical perspective. The clinical trial assessed host responses following consumption of HY7602-fermented deer antler extract, whereas the metabolomic analysis characterized the metabolic properties of the fermented food material itself. Through this product-level metabolic characterization, the present study aimed to clarify the biochemical features of HY7602-mediated probiotic fermentation and to characterize how fermentation alters the composition of deer antler extract.

After 12 weeks of supplementation, participants who consumed HY7602-fermented deer antler extract showed improvements in selected upper- and lower-limb functional outcomes. Compared with the placebo group, the intervention group exhibited greater increases in bilateral and unilateral hand grip strength. Right quadriceps strength also increased in the intervention group, whereas it decreased in the placebo group. In addition, chair-stand performance time in the Short Physical Performance Battery (SPPB) was reduced, indicating improved functional performance. Because hand grip strength, quadriceps strength, and chair-stand performance are widely used indicators of muscle function and lower-extremity performance in older adults, these findings suggest that HY7602-fermented deer antler extract may support selected measures of muscle-related function in adults with reduced muscle function [1, 3, 28, 33].

However, significant between-group differences were not observed for all outcome measures. The absence of a significant effect on left quadriceps strength may be partly related to side-to-side asymmetry in lower-limb strength. Previous studies have reported bilateral differences in knee extensor performance, although the direction and magnitude of dominant-limb effects appear to vary across populations and study conditions [34, 35]. Similarly, total SPPB score did not differ significantly between groups. This may reflect the relatively high baseline functional status of the participants. As a composite score ranging from 0 to 12, the SPPB can exhibit a ceiling effect in higher-functioning older adults, which may limit its sensitivity to detect subtle intervention-related changes [28, 36, 37]. This interpretation is consistent with the present results, in which chair-stand performance improved despite minimal change in total SPPB score. 4-m gait time also showed no significant between-group difference. Although serum superoxide dismutase (SOD) activity increased numerically in the intervention group, the between-group difference was not statistically significant. This finding suggests that circulating SOD activity may be a limited systemic marker for detecting changes in muscle function and that functional adaptations may be more closely related to local redox regulation within skeletal muscle tissue [38-40]. These results indicate that HY7602-fermented deer antler extract was associated with improvements in selected measures of muscle strength and physical performance, while also reflecting the heterogeneous responses commonly observed across functional outcome measures in intervention studies involving older adults [6, 41, 42].

Untargeted metabolomic profiling revealed clear compositional differences among the three production stages: extract (E), HY7602-supplemented extract (L), and fermented extract (F). Principal component analysis demonstrated stage-dependent separation, indicating that HY7602 addition and subsequent fermentation contributed substantially to overall metabolic variation. The intermediate distribution of the L group suggests that compositional changes began after HY7602 addition and became more pronounced following active fermentation. The largest metabolic divergence was observed between the E and F groups, supporting the interpretation that HY7602-mediated fermentation induced extensive biochemical remodeling at the product level.

Representative metabolite analysis further showed that fermentation-associated changes were not confined to a single metabolite class but involved multiple biochemical domains. N1-acetylspermidine and putrescine were associated with polyamine-related metabolism, whereas agmatine, histamine, and cadaverine reflected nitrogen- and amine-related metabolic processes. These amine-related metabolites are commonly observed in fermented food systems and are generally interpreted in the context of microbial nitrogen metabolism and fermentation-associated biochemical transformation [43, 44]. Changes were also observed in γ-glutamylglutamine, sn-glycero-3-phosphocholine, and aconitate, suggesting that HY7602-mediated fermentation affected γ-glutamyl peptide-related metabolism, phospholipid turnover, and central carbon metabolism.

Among the metabolites increased after fermentation, N1-acetylspermidine and putrescine are related to polyamine metabolism, which has been widely investigated in the context of cellular maintenance, aging biology, and skeletal muscle physiology [45, 46]. γ-Glutamylglutamine belongs to a group of metabolites associated with γ-glutamyl transfer reactions and glutathione-related metabolism [47, 48]. In addition, sn-glycero-3-phosphocholine and aconitate are linked to phospholipid metabolism and tricarboxylic acid cycle-related central carbon metabolism, respectively [49, 50]. These findings indicate that HY7602-mediated fermentation induced coordinated biochemical changes across polyamine metabolism, nitrogen and amine metabolism, γ-glutamyl peptide-related metabolism, phospholipid metabolism, and central carbon metabolism.

When considered together with previous in vitro and *in vivo* studies using HY7602-fermented deer antler, the present metabolomic findings help characterize the biochemical features of HY7602-mediated fermentation. Earlier studies primarily demonstrated biological effects, including improved endurance capacity, mitochondrial biogenesis, grip strength, stamina, muscle mass, muscle strength, attenuation of dexamethasone-induced muscle atrophy, and improvement of fatigue-related phenotypes [19-22]. However, those studies provided limited information on how the material itself was compositionally remodeled during fermentation. The present stage-resolved metabolomic analysis helps address this gap by showing that HY7602 addition and subsequent fermentation induced coordinated changes across multiple metabolic domains, including polyamine metabolism, nitrogen and amine metabolism, γ-glutamyl peptide-related metabolism, phospholipid metabolism, and central carbon metabolism. These findings do not establish a direct causal pathway between individual metabolites and clinical outcomes, but they provide an important product-level biochemical framework for interpreting fermentation-associated compositional changes in deer antler extract.

A notable feature of this study is the integration of a human intervention trial with product-level untargeted metabolomics using the same material administered to participants. The clinical trial demonstrated improvements in selected muscle-related outcomes, whereas the metabolomic analysis characterized the biochemical properties of the fermented product itself. Many metabolomic studies of fermented food materials rely primarily on endpoint comparisons between pre- and post-fermentation states, while stage-resolved metabolomic analyses that track compositional changes during fermentation remain relatively limited [23-25]. Therefore, the interpretation of fermentation-induced metabolic remodeling and its potential relevance to functional outcomes remains incomplete.

In this regard, the present study provides a stage-resolved description of metabolic changes occurring during HY7602-mediated fermentation. The metabolite groups identified in this study describe the biochemical landscape of the fermented material and provide a product-level framework for interpreting fermentation-associated biochemical characteristics. These data help explain how fermentation altered the composition of deer antler extract and provide a product-level biochemical context for interpreting previous preclinical findings and the present clinical results.

Future studies should incorporate targeted quantification of key metabolites, post-ingestion blood and urinary metabolomic analyses, absorption studies, gut microbiome and host-response profiling, and mediation-based statistical approaches. These approaches will be necessary to determine whether product-level metabolic remodeling is reflected in host metabolic responses and whether such responses are associated with improvements in muscle-related outcomes.

## Supplemental Materials

Supplementary data for this paper are available on-line only at http://jmb.or.kr.
