# Supplementary material for: Effect of HY7602-Fermented Deer Antler Extract on Selected Muscle-Related Outcomes and Fermentation-Associated Metabolomic Profiles
Source: J Microbiol Biotechnol. 2026 Jul 15;36:e2604018. doi: 10.4014/jmb.2604.04018 (PMC13442422; doi:10.4014/jmb.2604.04018)
Supplement: Supplementary file 1 [file jmb-36-e2604018-supple.pdf]

J. Microbiol. Biotechnol. 2026. 36: e2604018

https://doi.org/10.4014/jmb.2604.04018

Supplementary Tables

Table S1. Baseline characteristics of participants in the FA and placebo group.

Variable		FA Group (N=46)	Placebo Group (N=44)	*p*-value
Sex, n (%)	Male	6 (13.04)	8 (18.18)	0.5014 (C)
	Female	40 (86.96)	36 (81.82)	
Age, in years, mean ±SD		59.83 ± 5.86	59.30 ± 6.33	0.6807 (T)
Childbearing potential (females only), n (%)	No	5 (12.50)	3 (8.33)	0.7145 (F)
	Yes	35 (87.50)	33 (91.67)	
Alcohol consumption, n (%)	No	29 (63.04)	33 (75.00)	0.2207 (C)
	Yes	17 (36.96)	11 (25.00)	
Smoking status, n (%)	No	45 (97.83)	42 (95.45)	0.7416 (F)
	Quit <1 yr	0 (0.00)	0 (0.00)	
	Quit ≥1 yr	0 (0.00)	1 (2.27)	
	Current	1 (2.17)	1 (2.27)	
Exercise frequency, n (%)	None	10 (21.74)	10 (22.73)	0.3675 (C)
	1–2 times/ week	4 (8.70)	8 (18.18)	
	3–4 times/ week	12 (26.09)	10 (22.73)	
	5–6 times/ week	5 (10.87)	8 (18.18)	
	Daily	15 (32.61)	8 (18.18)	
Height, in cm, mean ± SD		158.04 ± 5.17	159.13 ± 6.34	0.3726 (T)
Weight, in kg, mean ± SD		57.08 ± 5.98	58.30 ± 6.55	0.3604 (T)
BMI, in kg/m², mean ± SD		22.88 ± 2.37	23.00 ± 1.94	0.7906 (T)

[C] Chi-square test and [F] Fisher's exact test were used for between-group comparisons of categorical variables; [T] two-sample t-test and [W] Wilcoxon rank-sum test were used for continuous variables.

Table S2. Within-group changes and between-group comparisons of outcome variables after 12 weeks of intervention.

Variables	FA group (n = 46)				Placebo group (n = 44)				p-value for between-group difference in change[b]
	Baseline	12 weeks	Change from baseline	Within-group p-value[a]	Baseline	12 weeks	Change from baseline	Within-group p-value[a]	
Bilateral Mean Hand Grip Strength (kg)	18.97 ± 0.57	22.84 ± 0.63	3.88 ± 0.47	<.0001***	20.90 ± 0.87	22.72 ± 0.90	1.81 ± 0.41	<.0001***	0.0017 (T)**
Left Hand Grip Strength (kg)	18.85 ± 0.60	22.26 ± 0.71	3.41 ± 0.55	<.0001***	20.55 ± 0.89	22.15 ± 0.95	1.60 ± 0.48	0.002**	0.0163 (T)*
Right Hand Grip Strength (kg)	19.08 ± 0.61	23.42 ± 0.59	4.34 ± 0.52	<.0001***	21.25 ± 0.87	23.34 ± 0.91	2.09 ± 0.45	<.0001***	0.0017 (T)**
Right Quadriceps Strength (N·m)	76.43 ± 2.95	80.93 ± 2.92	4.50 ± 1.98	0.0281**	82.83 ± 3.61	81.50 ± 3.51	-1.33 ± 2.11	0.5303	0.0467 (T)*
Left Quadriceps Strength (N·m)	76.77 ± 3.02	80.68 ± 3.27	3.92 ± 2.02	0.0584	82.59 ± 3.72	84.98 ± 3.63	2.39 ± 2.43	0.3310	0.6286 (T)
SPPB Chair Stand Time (s)	10.05 ± 0.38	8.79 ± 0.37	-1.26 ± 0.21	<.0001***	9.00 ± 0.39	8.50 ± 0.39	-0.50 ± 0.27	0.0698	0.0268 (T)*
SPPB total score	11.54 ± 0.11	11.74 ± 0.08	0.20 ± 0.07	0.0109*	11.77 ± 0.09	11.77 ± 0.08	0.00 ± 0.09	1.000	0.0714 (W)
SPPB 4-m gait time (s)	3.67 ± 0.07	3.47 ± 0.08	-0.20 ± 0.06	0.0024**	3.72 ± 0.07	3.55 ± 0.07	-0.17 ± 0.06	0.009**	0.6834 (T)
SPPB standing balance score	3.98 ± 0.02	4.00 ± 0.00	0.02 ± 0.02	0.3227	4.00 ± 0.00	4.00 ± 0.00	0.00 ± 0.00	-	-
SOD (U/mL)	14.60 ± 1.10	17.17 ± 1.23	2.56 ± 1.17	0.0335*	14.44 ± 1.31	14.57 ± 1.26	0.13 ± 0.60	0.8248	0.4480 (W)

Values are presented as mean ± SEM. Baseline and week 12 values are shown descriptively.

a Within-group comparisons between baseline and week 12 were performed using paired t-tests.

b Between-group comparisons for change from baseline were performed using independent two-tailed t-tests (T) or Wilcoxon rank-sum tests (W).

*p < .05, **p < .01, ***p < .001.

Table S3. **Summary of adverse events and safety outcomes.**

Parameters	**FA group (n=49)**	**Placebo group (n=51)**	***p*-value**
**Subjects with Adverse Events (AEs)**	12 (24.5%)	14 (27.5%)	0.7358[1)]
**Total Number of Adverse Events**	20	20	0.8712[1)]
**Severity**			
- Mild	18 (36.7%)	17 (33.3%)	0.7225[1)]
- Moderate	2 (4.1%)	3 (5.9%)	1.0000[2)]
- Severe	0 (0.0%)	0 (0.0%)	-
**Causality**			
- Definitely related	0 (0.0%)	0 (0.0%)	-
- Probably related	0 (0.0%)	0 (0.0%)	-
- Possibly related	1 (2.0%)	0 (0.0%)	0.4900[2)]
- Probably not related	13 (26.5%)	7 (13.7%)	0.1132[1)]
- Definitely not related	6 (12.2%)	13 (25.5%)	0.0905[1)]
- Unknown	0 (0.0%)	0 (0.0%)	-
**Discontinuation due to AEs**	0 (0.0%)	0 (0.0%)	-

Adverse events (AEs) and safety outcomes were compared between the HY-FA group (n = 49) and the placebo group (n = 51) over the 12-week intervention period. Data are presented as number of subjects with events and corresponding percentages (%). AE severity was classified as mild, moderate, or severe according to predefined clinical criteria. Causality was assessed by investigators and categorized as definitely related, probably related, possibly related, probably not related, definitely not related, or unknown.

Between-group comparisons for categorical variables were performed using the Chi-square test. When the expected frequency in any cell was less than 5, Fisher's exact test was applied. Statistical significance was defined as $p < 0.05$. No serious adverse events or discontinuations due to AEs were reported in either group.

[1)] *p*-values were calculated using the Chi-square test for comparison between the groups.

[2)] *p*-values were calculated using Fisher's exact test for small sample counts.
